# Systemic lupus erythematosus presenting with moyamoya and Guillain–Barré syndrome

**DOI:** 10.1093/rap/rkad077

**Published:** 2023-09-29

**Authors:** Amal Basnet

**Affiliations:** Rheumatology Department, ADK Hospital, Sosun Magu, Male, Maldives

Key message

- Plasma exchange is important in lupus for some rare immunological assaults non-responsive to immunosuppressive treatments.

Dear Editor, A 22-year-old female presented with complaints of fever on and off for 6 months, altered sensorium and limb weakness for 1 week. The weakness started with a sensation of heaviness in both feet, progressing rapidly to both upper and lower limbs in the next 5 days, leading to quadriparesis and a bed-bound state. Bowel and bladder sensations were intact. She had a history of malar rash, oral ulcers, photosensitivity and polyarthralgia. There was no history of recent flu-like symptoms, gastroenteritis, travel or immunization. There was no history of autoimmune diseases in family members. Neurological examinations revealed lateral rectus palsy of the left eye with left facial nerve palsy, power of 1/5 (Medical Research Council grading for muscle strength) in bilateral upper and lower limbs, with generalized hypotonia and generalized areflexia. She had truncal and respiratory muscle weakness, with wide fluctuations in pulse and blood pressure and increased sweating. Routine investigations, including a haemogram, liver and kidney function tests and serum electrolytes, were normal. Further work-up revealed positivity of ANA IF [1:320, 3+, nuclear homogeneous (AC-1)], an anti-dsDNA level of 105 IU/ml (reference <27 IU/ml), low complement components (low C3 and C4), negative aPL and proteinuria, with quantitative assessment of urine protein being 7 g/24 h. HIV, Hepatitis B and C were negative. Cerebrospinal fluid revealed albuminocytological dissociation, with absence of cells, a protein level of 140 mg/dl (reference range 18–58 mg/dl) and normal cerebrospinal fluid glucose. Nerve conduction revealed sensorimotor axonal polyneuropathy. A magnetic resonance angiogram of the brain revealed features of moyamoya syndrome (Fig. 1A). Renal biopsy was not done in view of the poor general condition of the patient. A diagnosis of lupus with neuropsychiatric involvement [Guillain–Barré syndrome (GBS), acute motor and sensory axonal neuropathy variant] with moyamoya syndrome and lupus nephritis was made. She was treated with pulse injection methylprednisolone (1 g daily for 3 days), followed by oral prednisolone (1 mg/kg). Injection of CYC was given according to the National Institutes of Health protocol. In view of her respiratory muscle weakness, mechanical ventilation was initiated. IVIG 2 g/kg was given over 5 days. Treatment with HCQ and angiotensin-converting enzyme inhibitor was started.

**Figure 1.**
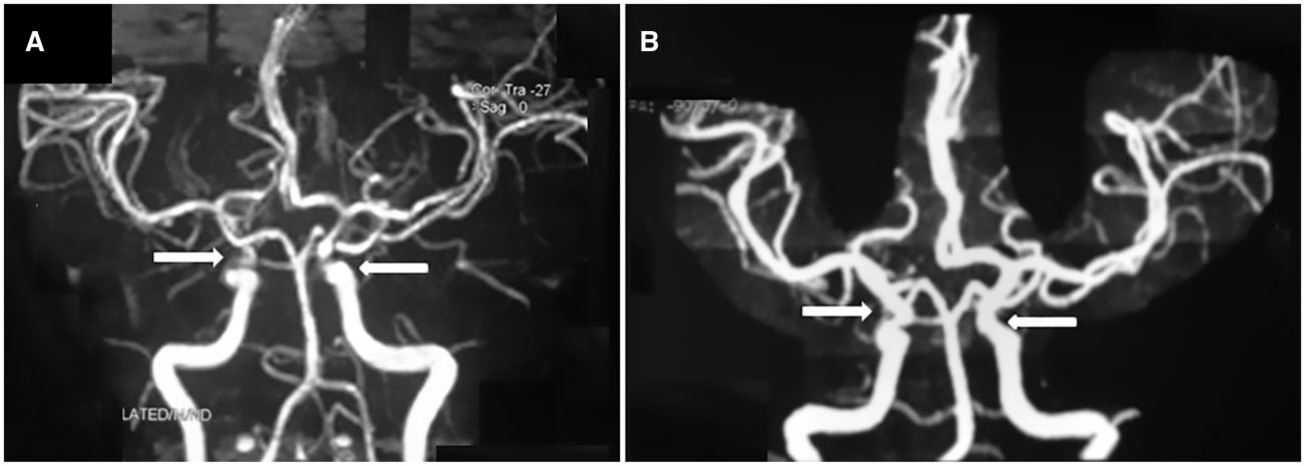
Magnetic resonance angiography of the brain showing moyamoya syndrome and reversal with treatment. (**A**) Attenuated bilateral supraclinoid internal carotid arteries (white arrows), with multiple collaterals and adequate distal intracranial reformation. (**B**) Revascularization of the previously attenuated supraclinoid artery (white arrows)

After 2 weeks, the patient showed no improvement in neurological status, and tracheostomy was done in anticipation of prolonged mechanical ventilation. Plasma exchange was initiated. The patient showed significant motor improvement after six cycles of plasma exchange, with weaning of mechanical ventilation and improvement in the motor power in the extremities to 3/5. Injection of CYC was continued monthly for 6 months. She had complete motor recovery at 3 months and angiographic resolution of moyamoya (Fig. 1B) with complete renal recovery at 6 months.

SLE is an autoimmune disease that can cause widespread immunological damage involving virtually any organ. GBS is the peripheral nervous system manifestation of neuropsychiatric lupus, with a reported prevalence of 0.6–1.7% [1]. Neuropsychiatric symptoms may pre-date the onset of lupus, occur concomitantly with lupus onset or may present later during the course of disease [2]. Both cell-mediated and humoral immune processes are assumed to have roles in the pathogenesis of GBS in lupus [3]. No definite treatment guidelines have been defined for treatment of GBS in lupus, but different combinations of CS, CYC, plasma exchange and IVIG have been used in the past, with complete recovery in most cases. Improvement in the symptoms is reported as early as 2 weeks after initiation of therapy, with complete resolution taking as long as 90 days [4].

Moyamoya syndrome is a cerebrovascular disorder characterized by progressive stenosis of intracranial internal carotid arteries and their proximal branches, leading to formation of collaterals and a ‘puff-of-smoke’ appearance on angiography [5]. Moyamoya syndrome is a rare presentation of lupus and has been reported in only a few case reports. The exact mechanism of moyamoya in lupus is unknown, but an immunological mechanism leading to CNS vasculitis that causes occlusion of the cerebral vessels is considered [6]. The inflammation leads to intimal smooth muscle cell hyperplasia along with endothelial cell proliferation, leading to formation of new vessels. Ultimately, this results in luminal stenosis and the formation of collaterals, leading to moyamoya changes on angiography [7]. Moyamoya syndrome can manifest as a cognitive disorder, mood disorder, acute confusional state, psychosis, seizures or stroke. The diagnosis is delayed in most cases because these are often the manifestation of lupus itself [8]. Treatment of lupus flare with an immunosuppressive reverted the vascular changes of moyamoya in our patient. The diagnosis of lupus nephritis was a clinical one, in view of active lupus, nephrotic range proteinuria and the response to immunosuppressants.

Isolated cases of lupus with GBS or lupus with moyamoya have been reported in the literature, with good treatment outcome. However, lupus presenting with GBS and moyamoya syndrome has never been reported. This is the first case of lupus with widespread immunological insult in the form of GBS, moyamoya and lupus nephritis, which was managed successfully with immunosuppressants and plasma exchange.

## Data Availability

The data underlying this article are available in the article and no additional source data are required.
